# Being implicated: on the fittingness of guilt and indignation over outcomes

**DOI:** 10.1007/s11098-021-01613-4

**Published:** 2021-03-03

**Authors:** Gunnar Björnsson

**Affiliations:** Department of Philosophy, Stockholm University, Stockholm, Sweden

**Keywords:** Moral responsibility, Reactive attitudes, Collective harm, Shared responsibility, Blame

## Abstract

When is it fitting for an agent to feel guilt over an outcome, and for others to be morally indignant with her over it? A popular answer requires that the outcome happened because of the agent, or that the agent was a cause of the outcome. This paper reviews some of what makes this causal-explanatory view attractive before turning to two kinds of problem cases: cases of collective harms and cases of fungible switching. These, it is argued, motivate a related but importantly different answer. What is required for fitting guilt and indignation is that the agent is relevantly *implicated* in that outcome: that the agent’s morally substandard responsiveness to reasons, or substandard caring, is relevantly involved in a normal explanation of it. This answer, it is further argued, makes sense because when an agent’s substandard caring is so involved, the outcome provides a lesson against such caring, a lesson central to the function of guilt and indignation.

## Introduction

When is it fitting for an agent to feel guilt over an outcome, and for others to be morally indignant with her over it? A popular answer requires that the outcome happened because of the agent, or that the agent was a cause of the outcome. In this paper, I motivate a related but importantly different answer. What is required is that the agent is relevantly *implicated* in that outcome: that the agent’s morally substandard responsiveness to reasons, or substandard caring, is relevantly involved in a normal explanation of it. This answer, I further argue, makes sense because when an agent’s substandard caring is so involved, the outcome provides a lesson against such caring, a lesson central to the function of guilt and indignation.

Throughout the paper, I’ll say an agent is *responsible* for something when they stand in the relation to it that makes moral guilt and indignation for it fitting. But “responsible” is multiply ambiguous, and my use is partly technical.^1^ The term could also be used to signify the relation required for an agent to deserve punishment for something, be liable to compensate for a harm resulting from it, or be liable to be harmed in defense of a threat posed by it. I set these latter forms of responsibility aside. It is at least conceptually possible that one can be subject to fair punishment or liable to defensive harm or compensatory demands even if all one’s actions have been justified and indignation would be misplaced.^2^

As stated, my concern is primarily with responsibility for *outcomes*, or states and events that are not constituted by the agent’s actions or responses or lack thereof to a situation: examples include an eroded friendship, a destroyed place of worship, or the deaths of hundreds of thousands of children from economic sanctions. (In line with ordinary moral thinking, but contrary to some luck-based skepticism, I will assume that we can be responsible for such outcomes in ways that make indignation and guilt over those outcomes fitting.^3^) I focus on outcomes because of the problems that they raise for theories of responsibility, but the account defended will be completely general.

Indignation over the injustice of some outcome need not target an agent. But when it does, it presupposes that the agent had (1) some capacity for understanding the values at stake, (2) some sort of self-control, (3) some relevant relation to the outcome, typically understood as a relation of control, and (4) knowledge or access to evidence of that relation. Since my focus here will be entirely on the third condition—*the outcome relation*—I will take for granted that other conditions are satisfied in the cases discussed unless otherwise stated. When I say that someone is the *fitting* target of indignation over something, I am saying that they satisfy the internal correctness conditions of such indignation, or the conditions that the systems responsible for producing indignation are supposed to track. I am not saying that that everyone or even anyone would be overall justified in nurturing or expressing such indignation.^4^

I begin in Sect. 2  by looking at problems for some natural accounts of the outcome relation. Section 3 suggests that these problems are avoided if we take the fittingness of guilt and indignation to be premised on a causal-explanatory requirement. Sections 4 and 5 introduce familiar counterexamples to the explanatory requirement: cases involving collective harms and “fungible switches”. Both kinds of cases suggest that the causal-explanatory requirement must be weakened. Section 6, finally, proposes such a weakening—it is enough that the agent’s substandard will is involved in a normal explanation of the outcome—and motivates it with reference to the nature of guilt and indignation.

## Closing in on the requirement of explanatory control

The idea that responsibility for outcomes involves a causal or explanatory component of some sort is widely accepted, though not universally so.^5^ One reason for resistance is that it faces the sort of counterexamples that will be discussed in Sects. 4 and 5. But another reason is that the causal-explanatory relation itself has been notoriously hard to pin down.^6^ Since some constraints on that relation will be of relevance when considering counterexamples, this section begins to motivate a requirement of explanatory control by noting how some related suggestions get cases wrong. We start with the tempting suggestion that outcome responsibility requires:

control whether: The agent had control over whether the outcome would materialize or not.^7^

For many cases, this seems to be exactly what makes the agent responsible for something. Consider:

*Wiley’s Window*: Suddenly, little Wiley picks up a pebble and throws it at a window, breaking it. A moment before, his big sister Suzy saw what was happening but did not prevent it.

Contrast two versions of this case:

*Arm’s Length*: Wiley was at arm’s length from Suzy, who could easily have stopped him.

*Out of Reach*: Wiley was out of reach, and Suzy had no way of stopping him.

Suppose that Suzy had no antecedent reason to think that Wiley would do anything like that, and so no reason to keep him within arm’s length. Then it seems that Suzy is a fitting target of (perhaps mild) indignation over the outcome in *Arm’s Length* but not in *Out of Reach* exactly because she controlled whether the outcome would happen in the former case, but not in the latter. (To avoid some tedious repetition, I will focus most discussion on the fittingness of indignation, returning to the fittingness of guilt in the last section).

However, control whether is in conflict with what most of us would want to say about other cases. Compare:

*Backup Billy*: Suzy mischievously throws a rock at the neighbor’s window, shattering it. Unbeknownst to Suzy, Billy was just about to throw a rock at the same window, but held his throw when Suzy’s rock hit. Had Suzy not thrown hers, Billy’s rock would have broken the window just as badly a couple of seconds later.

*Solo Suzy:* Like *Backup Billy*, but with Billy absent.

It seems that the neighbor can be indignant with Suzy over the sorry state of the window in both cases, even though Suzy had no control in *Backup Billy* over whether it would be in that state.^8^

In *Backup Billy*, it is natural to think that Suzy is responsible for the state of the window because it can be traced back to Suzy’s will through a process the earlier parts of which brought about the later parts: based on her desire to break the window, she formed an intention to throw a rock at it, which guided her to do so, which sent the rock flying towards the window, which made it hit the window, which broke the window. In light of this, one might think that rather than control whether, what is required for outcome responsibility is:

process control: The agent initiated or directed a causal process or series of processes leading to the outcome.

To capture our understanding of the example, we can think of processes as something like the “normal” unfolding of events according to law-like patterns that can be initiated or directed.^9^

If the outcome relation should be understood in terms of process control, this could explain why agents who lack control whether seem to be responsible for overdetermined outcomes in what is intuitively understood as:

backup cases: Cases where the agent had control over whether a certain process would lead to the outcome, but where the outcome would have been ensured by some other process had the first not done the job.

In line with its name, *Backup Billy* was such a case, but examples are easily multiplied, and can involve fitting gratitude as well as fitting indignation:

*Standby Steve*: Lara saw me struggling to find my destination and came to my aide. Unbeknownst to Lara, Steve also saw my predicament and would have helped if Lara hadn’t.

Because Lara’s intervention led me to find my way, she is responsible for my timely arrival in a way that might make gratitude for it fitting even if Steve’s presence ensured that I would have found my way without Lara’s help.

However, process control is clearly not *necessary* for outcome responsibility. Sometimes agents are responsible for outcomes in virtue of not having prevented a process leading to it, as in *Arm’s Length*. At other times, they can be so in virtue of having removed or prevented an obstacle to such a process without themselves getting involved in that process:

*Billy’s Board*: As Suzy was practicing her throw trying to hit a bullseye painted on a large board, Billy yanked away the board just as Suzy threw a rock. Because of this, the rock hit and broke the window that had otherwise been protected by the board.

Billy might clearly be responsible for the broken window, but he did nothing to initiate or direct the process from which it resulted: intuitively, he merely removed something that *would have* interfered with that process.

process control is also not *sufficient* for outcome control. Contrast the following:

*Redirection*: A runaway freight train is carrying dynamite. Unimpeded, it would continue on a track leading into the wilderness. Unfortunately for Rob, he has his handcar parked on that track, and it would be wrecked if hit by the train. To save the handcar, Rob can switch the train over to a track leading to a small town. He realizes that the dynamite might explode when the train crashes into the station, but prioritizes the handcar and makes the switch. Several people die when the cargo explodes at the station.

*Redundant Redirection*: Like *Redirection*, but unbeknownst to Rob, the track he thought led into the wilderness later remerges with the track leading to town. Had Rob not redirected the train, the deadly outcome would have been just the same.

In both cases, Rob can be blamed for willingly risking the lives of the townspeople to save his handcar. In both cases, Rob also gets involved in a process leading up to the explosion and the resulting deaths. But only in *Redirection* is he responsible for these deaths. Whereas it is fitting to be indignant with Rob over his willingness to risk people’s lives in *Redundant Redirection*, it isn’t fitting to be indignant with him over the outcome.^10^

*Redundant Redirection* belongs to a family of examples that are naturally understood as:

switching cases: Cases where the agent’s contribution explains why an already-evolving process led to the outcome in the way it did, but where the process would have led to the outcome as a matter of course without the agent’s contribution.

As with backup cases, examples are easily multiplied. Consider:

*Redundant Ride*: Moe the Mobster is on his way to beat up a shopkeeper who has refused to pay for “protection” and is about to leave town. Needing a ride there, Moe asks Joe, who is standing idly by his taxi car. Joe has overheard what Moe’s plans are, but wanting the money more than he cares about not contributing to Moe’s plans, he takes the ride. At the destination, Moe disappears into the shop where he breaks the shopkeeper’s arm. Had Joe not offered the ride, Moe would have asked some other taxi driver who would have gladly taken him there; though more scrupulous than Joe, the other drivers were ignorant of Moe’s mission. The outcome would have been the same.

The switching structure is clear here too: When Joe got involved, the process leading to the outcome—Moe’s acting on the intention to harm the shopkeeper—was already underway and would run to completion as a matter of course without Joe’s contribution; Joe only changed the exact way by which the process reached its destination. (Assume that there was nothing that Joe could reasonably have done to save the shopkeeper; crossing Moe would have meant risking his life.) Moreover, even though it is fitting to be indignant with Joe over his own contribution to Moe’s enterprise, being indignant with him over the outcome seems misplaced.^11^ Again, process control seems insufficient for fitting indignation.

## Explanatory control

The cases thus far seem to pull in conflicting directions, suggesting that counterfactual dependence sometimes matters (in switching cases and *Out of Reach*) and sometimes doesn’t (in backup cases), and suggesting that process control sometimes matters (in backup cases) but sometimes not (in switching cases and *Billy’s Board*). Importantly, this complexity closely mirrors a parallel complexity unearthed by the literature on causation and causal explanation. There, varieties of backup cases have provided counterexamples to theories understanding these relations in terms of counterfactual dependence, while switching cases have provided counterexamples to theories trying to understand them in terms of lawful sufficiency or processes.^12^ The parallel suggests a straightforward explanation for the complexity of the outcome relation, namely that it requires that some relevant aspect of the agent was a cause of the outcome, or that,

explanatory control: The outcome happened because of a morally relevant aspect of the agent.^13^

Applied to our cases from Sect. 2, the requirement of explanatory control yields the right verdicts. In cases where the agent seemed responsible for the outcome, there is also an explanatory connection: In *Backup Billy*, the window is in its sorry state because Suzy wasn’t properly concerned with the neighbor, even if Suzy didn’t control whether the window would be broken. In *Arm’s Length*, the window is broken because Suzy didn’t care enough about the neighbor to bother stopping her little brother, even if Suzy never interacted with the processes leading to the outcome; likewise for Billy in *Billy’s Board*. And in cases where the agent didn’t seem responsible, the explanatory connection is also missing: In *Redundant Redirection* (unlike in *Redirection*) the deaths didn’t happen because Rob cared insufficiently about the risks, and in *Redundant Ride*, the shopkeeper wasn’t assaulted because of Joe’s lack of concern for his safety, even if Rob and Joe got involved in the processes leading to these outcomes.

As stated, explanatory control leaves open what aspect of the agent should be a cause of or explain the outcome in order for moral indignation or guilt to be fitting. But the Strawsonian tradition provides what I think is the best answer, and one in line with what I just said: for these negative reactive attitudes to be fitting, the outcome must be due to the agent’s failure to care as can be morally required of her about what is morally important, or to her morally substandard quality of will.^14^ This suggests something like the following:

explanation responsibility: X is a fitting target of indignation and guilt over Y, and in that sense responsible for Y, if and only if Y is (i) morally bad and (ii) explained (in a normal way) by X’s morally substandard will.^15^

The exact nature of this condition depends on the nature of the *explanation * relation. For reasons of space, I offer no informative general account of that relation here. Instead I will rely on what I take to be pretty stable pre-theoretical intuitions about cases, and the sort of rough generalizations that I have offered about backup and switching cases.^16^ Hopefully, such intuitions have done enough to create a presumption for a condition of explanatory control and will do enough to motivate the modifications to that condition that I will propose in Sect. 6.

Before considering more cases, though, I want to address a general worry about this strategy, prior to worries one might have about claims about particular cases: Without recourse to a substantive informative account of the explanation relation, why should we think that a condition like explanatory control or explanation responsibility provides an *independent* condition on responsibility? Why not think that when our explanatory judgments about cases mirror our judgments about outcome responsibility, this is because our explanatory judgments track our responsibility judgments rather than the other way around?^17^ And this worry might seem to be supported by the fact that people are affected by normative considerations in making their causal judgments, standardly favoring factors that violate normative expectations when judging whether something caused a certain negative outcome: the person who broke the rules, or the mechanism that didn’t operate as it was supposed to.^18^

I will say three things about this worry. The first is that explanatory judgments and attributions of responsibility often come apart. Most obviously, we recognize that when a harm happens because of an agent that could not have foreseen the harm, the agent might not be responsible for it. More subtly, as illustrated in the next two sections, there are cases of overdetermined harms where someone is the fitting target of indignation over the harm even though it cannot be said to have happened because of them. The second is that while normative expectations do guide what we think of as causes of outcomes, this is true whether the judgments concern potentially responsible agents or inanimate mechanisms: the normative expectations at work fall short of judgments about responsibility for outcomes. The third is that standard explanatory judgments about cases like those discussed in Sect. 2 mirror judgments about cases without potentially responsible agents: indeed, such cases are prevalent in the causation literature. I cannot go through all our cases here and provide structurally equivalent non-agential cases, but for two simple illustrations, consider counterparts to our basic backup and switching cases:

*Non-Agential Backup*: A gust of wind blows a paper off my desk onto the ground. Had it not done that, the gust whipping through the room a couple of seconds later would have done the same.

*Non-Agential Switch*: Like *Redundant Redirection*, but a falling branch switches the train over to the wilderness track.

Just as it seems true about *Backup Billy* that the neighbor’s window is broken because of Suzy’s mischievousness, it seems true in *Non-Agential Backup* that the sheet of paper is on the ground because of the first gust of wind. And just as it seems false in *Redundant Redirection* that the deaths at the station happened because of Rob’s lack of concern with the townspeople, it seems false in *Non-Agential Switch* that they happened because of the falling branch.

I think that the cases considered thus far provides strong prima facie support for a requirement of explanatory control. In the following two sections, however, we will consider how it fails to account for the fittingness of indignation over some *collective harms* and some harms due to what I will call *fungible switches*.

## Fitting indignation and collective harms

Collective harms are harms that are explained by the actions or inactions of more than one individual but cannot be said to be explained by the contribution of any one of those individuals.^19^ Here is a typical case bringing out the particular problem that concerns us, modelled on cases in Held (1970):

*Demise*: As a man’s house collapses during an earthquake, a falling beam crushes his leg and pins him to the floor. Unaware of each other, three passersby hear his desperate but fading cries for help, but no one approaches to see what the problem is: the site looks dirty and they are eager to escape the unpleasant weather. Had at least two of them heeded the man’s calls, they would have been able to move the beam and bring him to hospital before his demise. But had only one done so, she would have been unable to move the beam, or to get others to help.

Assume that each of the passersby is a fitting target of indignation for not responding to the cries for help. Given what she knew, she just lacked sufficiently weighty reasons not to. Further assume that if they all had responded adequately, they would have realized the problem, teamed up, and saved the man. Then it seems that:

indignation over demise: The passersby are fitting targets of indignation over the man’s demise.

But it isn’t true about any one of them that:

explanation of demise: The man expired under the beam because that passerby didn’t care enough about the values at stake.^20^

This looks like a problem for explanation responsibility. Moreover, *Demise* is representative for a wide variety of cases, involving both small groups, as in *Demise*, and very large groups, as in:

*Climate Threat*: We would not have been facing the threat of climate catastrophe if, during the last 30 years, enough people who were aware of the problem had taken steps to promote prevention of it and refrained from supporting and engaging in actions particularly prone to promote greenhouse gas emissions.

Assume that it is fitting to be (perhaps mildly) indignant with these individuals over their individual contributions to the threat and their various failures to promote prevention.^21^ Under that assumption, I think that most of us would also want to say that the group of people who were aware of the problem but did nothing are fitting targets of indignation over the fact that we are now facing a very severe global threat. But, again, this is in conflict with explanation responsibility, as it is false of just about every member of that group that we are facing a severe threat because of that member’s morally substandard will.

One response to the lack of individual control in collective harm cases is to say that *the group* is responsible for the outcome and a proper target of indignation. That claim is in line with explanation responsibility, as it seems true that the man in *Demise* expired because the substandard wills of three passersby, and true that we are facing a very severe global threat because those aware of the climate risks did not take them as seriously as they should have.^22^ But it fails to capture the thought that.

implicated individuals: Contributors to collective harms are implicated in those harms in ways that make it fitting to be indignant with them over these outcomes and for them to feel guilt.

It is not that the indignation or guilt is directed at the groups as entities existing over and above the individual members, but rather that it is directed at the members qua members of such groups. In line with this thought, a number of people (me included) have suggested that the agents are *jointly* responsible for the outcome, or that they *share* responsibility, or some such.^23^ But if indignation over an outcome can be fittingly directed at a member of a group in virtue of *the group’s* explanatory control over the outcome and in the absence of *the member’s* control, then explanation responsibility is too strict a demand.

explanation responsibility needs to be weakened, but how? A tempting first proposal would be to merely require that the agent culpably contributed to the causal process leading to the harm. But that requirement is too weak, as illustrated by *At Arm’s Length* and *Billy’s Board*. It is also too strong, as illustrated by *Redundant Redirection* and *Redundant Ride*.^24^

An alternative suggestion requires that the agent culpably contributed part of a cause of the outcome.^25^ But that doesn’t seem right either. Consider:

*Algae Bloom*: A massive algae bloom produced large amounts of toxin, enough to kill all the fish in a lake several times over. During the bloom, Adam culpably disposed of paint solvent in the lake, solvent he knew contained a toxin. Though the small amount of toxin that he contributed happened to be the very same that was produced by the algae and thus was part of what killed the fish, it made no difference to the outcome and would have been harmless on its own.^26^

Though the fish died because of the toxin in the lake, and though Adam culpably contributed to that toxin in a way that makes indignation over his recklessness fitting, it does not seem fitting to be indignant with Adam over the death of the fish.

One might think that what distinguishes *Demise* and *Climate Threat* from *Algae Bloom* is that the agent culpably contributes to a cause of the harm that is due to *a group of agents*. But that also does not seem right:

*Innocent Polluters*: Having been erroneously told by what is normally a very reliable source that a boat paint solvent was non-toxic, a large group of people disposed of it in the lake, enough to kill the fish in the lake many times over. At the same time, Adam culpably disposed of the same paint solvent in the lake, knowing that it contained a toxin. Though the small amount of toxin that he contributed was part of what killed the fish, it made no difference to the outcome and would have been harmless on its own.^27^

In this case, like in *Algae Bloom*, Adam seems to be a fitting target for indignation over his reckless handling of the solvent, but not over the death of the fish. This suggest that what is required is that all members of a relevant group *culpably* contributed to the cause of the harm, as in *Demise* and *Climate Threat*. Buy why should the culpability of other agents affect whether one is fittingly indignant with this one agent over the outcome? The reason, I will suggest in Sect. 6, is that when a collective harm happened because each of a group of agents failed to live up to demands on their will, then each agent’s substandard will is relevantly involved in a normal explanation of the outcome. Because of the nature of guilt and indignation, this sort of involvement is exactly what is required.

First, though, we will consider another kind of problem case.

## Fitting indignation and fungible switches

For the next sort of problem for explanation responsibility and the explanatory control requirement, consider a series of three related cases, starting with.

*Dealer*: Doug, a drug dealer, recognizes Adele, an addict, as she is heading to the supermarket. He approaches and although she had planned to stay off drugs, she buys from him. When Adele later takes the dangerous drug, YXS, it triggers a psychosis during which she drives off a cliff and dies.

Assume that Doug had neither good reasons nor good excuses for selling Adele a dangerous drug, and that it is thus fitting to be indignant with Doug over this action. Then it also seems fitting to be indignant with him over Adele’s death.^28^ This is in line with explanation responsibility, as Adele died because Doug didn’t care as morality demands about the values at stake. Contrast this case with:

*Dealer v. Pharmacy*: As in *Dealer*, but Adele had a legitimate prescription for YXS and was heading to the pharmacy when Doug approached. Being offered the very same drug at a lower price, she buys it from him. When she later takes the drug, it triggers a psychosis during which she drives off a cliff and dies, just as in *Dealer*. If Doug had not been willing to sell the dangerous drug, she would have pursued her original plan and had the pharmacy fill her prescription; the outcome would have been the same.

This is an ordinary switching case, like *Redundant Redirection* and *Redundant Ride*. A process—Adele’s intending to get the drug—was under way and would, as a matter of course, lead to Adele getting the drug, having the psychosis, and killing herself by driving over a cliff; what Doug did was merely to change the exact way the process ran to completion. Even assuming that it is just as fitting to be indignant with Doug over his act of selling YXS to Adele as it was in *Dealer*, it now seems unfitting to be indignant with him over her death. This, again, is in line with explanation responsibility, as it is false that Adele died because of Doug’s substandard will.

But now consider our third case:

*Dealer v. Dealers*: Like *Dealer v. Pharmacy*, but Adele had no prescription and was heading to her regular dealers to buy YXS when Doug approached. Had Doug not sold her the drug, the outcome would have been the same: she would have followed her original plan, bought the drug from one of the other dealers, and died driving off a cliff.

Like *Dealer v. Pharmacy*, this is a switching case, and here too it seems false that.

explanation of death: Adele died because of Doug’s substandard will.^29^

But unlike in *Dealer v. Pharmacy*, and contrary to what one should expect given explanation responsibility, it seems to me that.

indignation over death: Doug is a fitting target of indignation over Adele’s death.

For Adele would not have successfully executed her plan to get the drug if everyone had just cared as can be demanded about the values at stake.

More specifically, here is what I think makes the difference between *Dealer v. Pharmacy* and *Dealer v. Dealers*: though it is true about both cases that Adele’s death would have happened without Doug’s disregard for the values at stake, only in *Dealer v. Dealers* did the process require that *someone* disregarded these values in the way Doug did. In virtue of this, *Dealer v. Dealers* is a case of “fungible switching”:

fungible switching cases: Cases where (i) the agent’s culpable contribution explains why an already evolving process led to the outcome in the way it did, (ii) for the process to lead to that outcome as a matter of course, someone’s culpable contribution was required, and (iii) the agent’s contribution was fungible, and someone else would have made the required culpable contribution had the agent abstained.^30^

So understood, fungible switching cases are legion: A weapons manufacturer hires an engineer to design a part for a land mine that ends up killing and maiming civilians, but numerous other engineers were willing and able to supply that knowledge should this engineer refrain. A fascist political party hires a copy writer for an advertisement campaign when numerous other copy writers would have been willing and capable of doing it instead. A concentration camp hires a guard for a job that numerous others would have been willing to do. In each of these cases, a harm results from an overall process and the availability of agents willing to culpably contribute to that process. And each case provides a counterexample to explanation responsibility: the agent enlisted in the project is a fitting target of indignation over the harmful outcome, but it is false that the harms take place because of their substandard will.

Why, though, does it matter that someone’s culpable contribution was needed for the process to yield the outcome? The reason, I suggest, is that the substandard will of the agent who actually contributes is then involved in what explains the outcome. Even if it isn’t true of *Dealer v. Dealers* that Adele died because of Doug’s disregard for the values at stake, it is true that.

existential explanation of death: Adele died because she found someone who disregarded the relevant values enough to sell her the drug.

Since that someone was Doug, his substandard will is involved in an explanation of the outcome. And the same goes for other cases of fungible switching: they are cases where the outcome takes place because the process at work enlists the contribution of someone willing to ignore certain values.

## Reactive attitudes and outcomes

Cases of collective harms and fungible switches provide counterexamples to explanation responsibility. However, I have suggested that the troublesome examples could be handled by something like the following modification of explanation responsibility:

implication responsibility: X is a fitting target of moral guilt and indignation over Y if and only if Y is morally bad and X’s morally substandard will is involved in a normal explanation of Y—if X is “implicated” in Y.

But more needs to be said about what it is for one’s substandard will to be “involved” in a “normal” explanation. The key to understanding this, I will suggest, lies in some central features of moral indignation and guilt.

The kind of moral indignation that concerns us here has both a target and an object: we are indignant *with* someone *over* something—some action, reaction, omission of theirs, or some outcome. Likewise, the feeling of moral guilt over something targets oneself in relation to the object of guilt.

Both sorts of reaction are based on two interrelated evaluations: an evaluation of the object of the emotion as morally bad in some way (instrumentally or non-instrumentally), and an evaluation of the agent’s will as falling short of what morality demands of her. Each of these evaluations can have emotional repercussions on its own: sadness, pain, horror, disappointment, contempt, or shame. What makes moral indignation and guilt *over something* special, however, is the relation between the attitudes towards the object in question and the attitudes towards the substandard will.

To understand that relation, it is important to understand that demands on the will are demands that we *care* about things because these things are important: demands that we be alert to what makes it go well with these things and that we adjust our responses accordingly.

This matters, first, because caring has a psychological function: to protect and promote the object of care. This means that it has *functionally normal* ways of operating,^31^ and thus that there are normal ways in which caring as demanded about something can make it go well with it: we notice and act skillfully on possibilities to promote or protect what we care about. By extension, there are normal ways in which things go badly with that object because of failures to care as required, as required by implication responsibility.

It matters, secondly, because caring about things as morality requires is a *skill* the function of which is to adequately promote and protect certain values. Like other skills, it is subject to feedback: when shortcomings are involved in bad outcomes, this can provide direct corrective lessons for the agent or for others learning from her mistakes. Accordingly, moral guilt and indignation seem to involve the idea that their objects provide direct lessons about the importance of caring as required. This aspect is often salient in guilt, which paradigmatically involves not only reactions to the object of guilt and to one’s own shortcoming in light of that object, but also the sense that the object of guilt highlights the importance of not falling short in that way. It is also salient in paradigmatic instances of indignation targeted at someone over something, which seek recognition of this lesson in the guilty party. The suggestion now is that for one’s substandard caring to be involved in a normal explanation of the outcome in the way of implication responsibility is for it to be involved in such a way that the outcome provides a direct corrective lesson.

What is it to be so involved? Here I can offer no general account. Instead, I will mention three ways in which bad substandard responsiveness to the circumstances can explain outcomes in a way that grounds direct feedback. I will also illustrate these by non-moral examples involving the substandard responsiveness of only one agent:

1. *A single instance of substandard responsiveness explains a bad outcome*: Kit broke a glass while doing dishes because he was insufficiently concerned with its fragility. That the glass broke because of his carelessness on the occasion provides a lesson against such carelessness.
2. *Several instances of substandard responsiveness jointly explain a bad outcome:* Because Kim had on numerous occasions imprudently disregarded the need for an economic buffer, she cannot afford repairs when her car breaks down. Because the repairs would be very expensive, it isn’t true about any one instance of imprudence that her predicament is due to that instance, as she would have been short even if she had saved up on that occasion. But the outcome nevertheless seems to provide a corrective lesson against such imprudence.
3. *The existence of some substandard responsiveness explains a bad outcome*: The kids had planned to pillage their parents’ candy stash behind the back of Alex, the babysitter, and did so at a time T, when his attention was elsewhere. If Alex had been attentive at T, however, they would just have had to wait a few minutes, as his attention was frequently on other things than the children. Given this, it is false that the candy was gone when the parents came home because Alex was inattentive at T. But it is nevertheless true that it was gone because there was a moment when he was inattentive. And because of this, the role of his inattention at T in the disappearance of the candy provides a lesson against such inattention.

Summing up, the suggestion is that paradigm cases of moral guilt and indignation consist in typical emotional reactions to (1) a bad object, (2) one or more substandard wills, and (3) the involvement of the latter in a normal explanation of the former.^32^ When we think that guilt and indignation over the outcome are fitting in some of the cases that we have considered but not in others, it is these sorts of reactions that we imagine. It should be clear how the various cases that we have looked at exemplify the three types of involvement in explanations of outcomes listed above: *Arm’s Length*, *Backup Billy*, *Billy’s Board*, *Redirection*, and *Dealer* all exemplify the first type, while collective harm cases like *Demise* and *Climate Threat* exemplify the second, and fungible switching cases like *Dealer v. Dealers* exemplify the third. Independently of type of involvement, what they all have in common is that the outcomes provide lessons against the substandard caring exemplified by the agent.

I have argued that guilt and indignation over an outcome can fittingly target an agent even if the outcome did not happen because of the agent’s substandard will. For an agent to be suitably implicated in the outcome, it is enough that the agent’s substandard will is *involved* in a normal explanation of it. If this is correct, it leaves a number of further questions: If someone joins a team engaged in genocide but is never called on to contribute, is that person part of a group whose substandard wills explain the outcome? If someone commits an atrocity expressive of a lack of concern with human life and I share that lack of concern, can I be the fitting target of indignation over the atrocity because of the *kind* of substandard will that I harbor, even if it isn’t due to my token substandard will? Are different degrees of guilt and indignation fitting depending on how an agent is implicated in an explanation of harm?

Though the general picture of guilt and indignation presented here is suggestive of answers to these questions, further discussion will have to wait. Instead, let me end by noting this: Many who follow Strawson in understanding responsibility in terms of reactive attitudes have taken these attitudes as responding to agents’ qualities of will. But in contrast to this, our practices of holding one another responsible are often very explicitly about outcomes. The present account helps to makes sense not only of how guilt and indignation can be deeply involved in the outcomes that flow from the agent’s will, but also of the range of outcomes that these emotions can take.

## Footnotes

1 Though technical, the use follows a tradition from Peter Strawson’s (1962) “Freedom and Resentment” of understanding responsibility as a precondition for reactive attitudes, and in particular for guilt and indignation. For simplicity, I refrain here from discussing other interestingly related reactive attitudes, including resentment.

2 Cf. Jeff McMahan’s (2005) suggestion that liability to defensive harm requires a form of responsibility for the threat, but not the sort of involvement that makes guilt and indignation fitting. One might disagree with McMahan’s suggestion, but it is not conceptually incoherent. For some recent discussions of what sort of involvement is required for liability to defensive harm, see e.g. Kutz (2007), Frowe (2014), Haque (2017).

3 For luck-based skepticism, see e.g. Enoch and Marmor (2007). For my account of why intuitions driving such skepticism shouldn’t be trusted, see Björnsson and Persson (2012).

4 For the classical discussion of fittingness, see D'Arms and Jacobson (2000).

5 For examples of how causal-explanatory conditions can be incorporated, see e.g. Fischer and Ravizza (1998, ch. 4), Sartorio (2007, 2016). For my own previous suggestion, see Björnsson (2017a); Björnsson and Persson (2012).

6 For an excellent overview of many central problems, see Paul and Hall (2013).

7 Cf. what John Fischer (1994) calls *regulative* control, and the *sine qua non* condition in some legal frameworks. control whether also obviously corresponds to the simple counterfactual theory of causation, and is subject to the same counterexamples (see e.g. Lewis 1973; Paul and Hall 2013), as we shall soon see.

8 *Backup Billy* illustrates the same point as *Missile 2* in Fischer and Ravizza (1998). Cf. Lewis’s (1986) backup assassin case.

9 This condition corresponds to accounts trying to understand causation in terms of processes or suitably constrained relations of lawful sufficiency, as in Mackie (1974) or (in a very different way) Dowe (1995).

10 Analogous cases are employed in van Inwagen (1983), Fischer and Ravizza (1998).

11 I’m assuming here that it might be morally problematic to get involved in moral wrongs even when one cannot reasonably affect whether they are committed or what their consequences will be. This is a controversial assumption. For articulation of skepticism for cases where agents cannot significantly affect some grand outcome, see especially Sinnott-Armstrong (2005), Kingston and Sinnott-Armstrong (2018). I defend the assumption in Björnsson (2021); cf. Nefsky (2016). As noted in the introduction, to deny that Joe is the fitting target of guilt or indignation over the beating or the resulting injuries (and so to deny that he is in that sense responsible) is not to deny that Joe is liable to bear restorative costs related to the injuries (and in that sense bears responsibility for it) or that he would be liable to defensive harm in attempts to avert the injuries (see e.g. Frowe 2019).

12 For a recent overview of many of these complexities, see e.g. Paul and Hall (2013), cf. Björnsson (2007).

13 I have formulated the requirement in terms of explanation rather than causation, as the latter is often understood in too restrictive a way. In *Arm’s Length*, for example, it seems clearly right that the window shattered because Suzy didn’t bother to stop her little brother but not clearly right that her lack of concern caused it to shatter. Similarly, one might think that lack of antibodies explains someone’s covid-19 illness while denying that the lack of antibodies caused the illness—the virus did! However, a requirement that some aspect of the agent be *a* cause of the outcome might be fine: it is more natural to say that Suzy’s indifference was a cause of the shattering, and that the lack of antibodies was a cause of the illness. (Cf. Sartorio’s (2007) formulation of a transmission of responsibility principle.).

14 See e.g. Strawson (1962); McKenna (2005, 2012); Arpaly (2006); Arpaly and Schroeder (2014).

15 I have previously defended a suggestion of this form. See Björnsson (2017b); Björnsson and Persson (2013, 2012).

16 Given that none of the best-known systematic theories of causation or causal explanation (e.g. counterfactual, INUS/NESS, and process theories) does a great job accounting for such intuitions (for difficulties, see e.g. Paul and Hall (2013)), one might worry about trusting these intuitions. However, I argue elsewhere (Björnsson 2007) that explanatory intuitions can be made sense of on a natural systematic development of the view that causal explanation is a secondary relation, having its unity in the way it can serve instrumental reasoning (for an early sketch of such an account, see Menzies and Price (1993)).

17 I thank Helen Frowe, Massimo Renzo and an anonymous referee for pressing me on this.

18 For discussion of the view that causal or explanatory judgments are partly normative, see Sartorio (2007): 754ff. For evidence that causal-explanatory judgments generally are sensitive to normative assumptions, see e.g. Knobe and Hitchcock 2009.

19 Problems of responsibility for collective harms have been widely discussed (see e.g. Held 1970; May 1992; Kutz 2000; Isaacs 2011; for a useful overview, see Smiley 2017). But not all is relevant for our current concern with the relation between agents and outcomes that can make indignation with those agents over the outcomes fitting. Much of the general debate concerns other questions, such as the relations required to owe compensation for harms or be liable to defensive harm. Much of the discussion also concerns cases involving cooperative groups and features specific to these, but the collective harm problem that concerns me here arises equally for harms resulting from the uncoordinated actions or inactions of numerous agents and is not essentially liked to cooperation and joint action (Björnsson 2011). Finally, some of the discussions are concerned with when one agent is blameworthy or liable based on their complicity in the wrongs committed by other agents. By contrast, agents in the cases discussed in this section are more saliently implicated in collective harms than complicit in the wrongs of other agents.

20 The explanatory judgments here mirror those of non-agential cases with the same structure: Suppose that a barn door has three latches, that two would have been required to keep it in place when pushed by the heifers, but that all latches were open one day and the door swung open when pushed. It seems correct that it swung open because the latches were open, but false about any one latch that the door swung open because that latch was open.

21 As noted in n. 11, this is a controversial assumption.

22 The group arguably lacks any will of its own, but as I argue elsewhere (Björnsson), explanation responsibility can be generalized to cover both individuals and groups.

23 For some very different accounts, see e.g. May (1992); Kutz (2000); Sartorio (2004); Björnsson (2011).

24 Cf. Heinaman’s (1986) suggestion that the agent must be “among the causes of” or “part of a full explanation of” the outcome. For criticism, see Fischer and Ravizza (1998).

25 For a closely related view, see Sartorio (2004).

26 *Algae Bloom* does the same work as *Adam’s Lake* in Björnsson (2011).

27 *Innocent Polluters* does the same work as *Partly Excused Helplessness* in Björnsson.

28 I’m assuming that one can be indignant with Doug over Adele’s death while thinking that Adele herself was also responsible for the outcome, and perhaps to a higher degree.

29 One might think that by illegally selling drugs, Doug has indirectly contributed to a system supporting both addictions and the illegal selling of drugs, and that he is thus indirectly responsible for the outcome. To avoid this interpretation, assume that it was Doug’s first deal.

30 The idea that agents can be responsible for outcomes in fungible switching cases is closely related to the suggestion that an agent is responsible for an outcome if they culpably provided something required for that outcome, even if it wasn’t required that they provided it (cf. Sartorio 2016). However, the latter principle gets a case like *Innocent Polluters* wrong, and would similarly yield what I take to be the wrong result for *Dealer v. Pharmacy*. (By contrast, a related view about liability for defensive harm (rather than fitting indignation) seems correct for culpable contributions to wrongful threats. See Frowe 2019).

31 For one way of understanding the relevant notion of normality, see Millikan (1984).

32 Elsewhere, I discuss other important aspects of these reactions. Particularly important are guilt’s tendency towards submission and efforts to mend the situation and indignation’s tendency to demand these things of its target. But these tendencies are shaped by the lessons identified by guilt and indignation, and so by the relevant kinds of involvement.
